# The Frequency of Medically Compromised Patients in Endodontic Offices in Iran

**Published:** 2013-05-01

**Authors:** Masoud Parirokh, Mohammad Jafar Eghbal, Jamileh Ghoddusi, Shahla Kakoei, Ali Akbar Haghdoost, Sina Kakooei

**Affiliations:** 1 Oral and Dental Diseases Research Center, Dental School, Kerman University of Medical Sciences, Kerman, Iran; 2 Iranian Center for Endodontic Research, Research Institute of Dental Sciences, Shahid Beheshti University of Medical Sciences, Tehran, Iran; 3 Department of Endodontic, Dental School, Mashhad University of Medical Sciences, Mashhad, Iran; 4 Research Center for Modeling in Health, Kerman University of Medical Sciences, Kerman, Iran

**Keywords:** Endodontics, Medically compromised, Systemic diseases

## Abstract

**Introduction:**

As the result of epidemiological transition and aging of Iranian population, the frequencies of systemic diseases among patients in of need endodontic treatment has increased, especially within developed cities. However, there have been no concise reports of systemic diseases in Iranian patients. Based on this need, the present investigation was conducted to assess the frequency of systemic disease among patients referred to endodontic private practice in three main cities in Iran.

**Materials and Methods:**

In a retrospective study, the frequency of systematic diseases were abstracted from the health records of patients who were referred to three private practices limited to endodontics in Kerman, Mashhad, and Tehran between 1994 to 2011.

**Results:**

Overall, 15,413 records of patients were assessed. The patterns of systematic diseases among endodontic patients in these three cities were different. The overall frequency of systemic disease in Kerman was significantly higher than two other cities (Kerman: 55.03%, Mashhad: 24.32%, Tehran: 22.16%; P<0.001). The most commonly occurring diseases were cardiac disease, hypertension, allergy and neurological disorders.

**Conclusion:**

Since the number of endodontic patients with systematic diseases is considerably significant and varied, special training and educations for treatment of medically compromised patient should be considered at both post- and undergraduate training.

## 1. Introduction

Patients increasing awareness of the importance of dental and oral health resulted in increasing the number of patients seeking advanced dental health care (1). Since some of the patients referred to receive dental treatment may have systemic disorders, dentist should have enough information to manage these medically compromised patients (2, 3).

In order to provide reasonable dental treatment for all patients it is necessary to obtain medical history prior to dental treatment (4). For medically compromised patients some modifications in treatment plan or material may be required in order to prevent probable interaction with their current medications or systemic health stability (1, 2, 4). Unfortunately, in Iran there is no general agreement regarding the time/syllabus needed to teach various medical conditions. Rationally, more common systematic diseases should be emphasized and taught during both undergraduate and postgraduate dental training. Thus investigating the frequency of diseases in patient who referred to dental offices may at least provide some information regarding the frequency of systemic diseases that might be encountered during daily practice.

Several investigations have reported frequency of systemic diseases among dental patients in various countries including Australia, Thailand, Saudi Arabia, USA, Jordan, the Netherlands, the West Indies, and Ireland (5-13), but the reported frequencies of systemic diseases among these countries have not been similar. It is necessary to investigate prevalence of systemic diseases among referring patients in various geographical places.

Based on our search in two main data bases (PubMed, and Scopus) no investigations has been found about the frequency of systemic diseases among patients who referred to either general dental offices or any specialist in Iran. The aim of the present study was to investigate the frequency of systemic diseases among the patients who referred to the offices limited to endodontic practice in three main cities of Iran.

## 2. Material and Methods

In this retrospective study medical records of patients who referred to three private practices limited to endodontics in Kerman, Mashhad, and Tehran were collected. The city of Kerman is the center of the largest province in southeast Iran; the estimated population for 2010 was 570,000. Mashhad is the center of Khorasan Razavi province in north east of Iran and the second largest city of the country; the estimated population in 2010 was three millions. Tehran, the capital of Iran, located north of the center of the country has an estimated population of ~10 million in 2010.

These cities were selected because they are in three different geographic positions and the selected practices had thorough medical history forms and provided questionnaires regarding patients’ health status. All of practitioners in the selected offices in these three cities have trained their reception nurse for demanding more information from the patients if they have marked any systemic conditions when filling out the questionnaire and the reception nurses have to inform the practitioner regarding specific systemic condition of the patients; finally, practitioners should review the questionnaire as they are legally responsible.

All of the endodontic practitioners were seriously committed to ask more details about their systemic diseases from their physician to see whether modification in dental treatment plan was required. In addition all these three offices have been active for more than 10 years. All documents which contained a self-completed health questionnaire were reviewed. The list of extracted data from each patient’s document contained demographic information such as age, sex, history of cardiac disease, hypertension, respiratory disease, allergies, asthma, kidney disease, hepatitis, pregnancy, rheumatic fever, neurological diseases, gastric disorder, diabetes and patients with hematologic disorders. Only documents that have been completely filled by the patients were included in the present study.

The frequency of every systematic diseases and their overall frequency were computed and classified according to the city. These frequencies were compared using chi-square test with Bonferroni correction. P-value less than 0.05 were considered significant.

## 3. Results

Overall, 15413 documents from three different cities consist of Kerman (*n*=5911), Mashhad (*n*=4589), and Tehran (*n*=4913) had valid records (Table 1).

**Table 1.** The frequency of systemic diseases among patients referred to three different cities of Tehran, Mashhad, and Kerman

		Kerman	Mashhad	Tehran
**Cardiac Disease**	No	4824	4407	4675
	Yes	1087	182	220
**Rheumatic Fever**	No	5423	4558	4880
	Yes	488	31	33
**Renal disease**	No	5701	4479	4803
	Yes	210	110	102
**Asthma**	No	5423	4553	4836
	Yes	488	36	71
**Hepatitis**	No	5374	4528	4835
	Yes	537	61	71
**Diabetes**	No	5620	4502	4822
	Yes	291	87	89
**Neurologic Disease**	No	5016	4447	4847
	Yes	895	142	62
**Hypertension**	No	5470	4218	4617
	Yes	441	371	287
**Hematologic Disease**	No	5847	4555	4873
	Yes	64	34	33
**Gastric Disease**	No	5479	4576	4913
	Yes	432	13	0
**Allergy**	No	5526	4370	4546
	Yes	385	219	367
**Other Diseases**	No	5711	4348	4614
	Yes	200	241	299
**Medication**	No	5176	4051	3710
	Yes	735	538	1203

**Figure 1.**
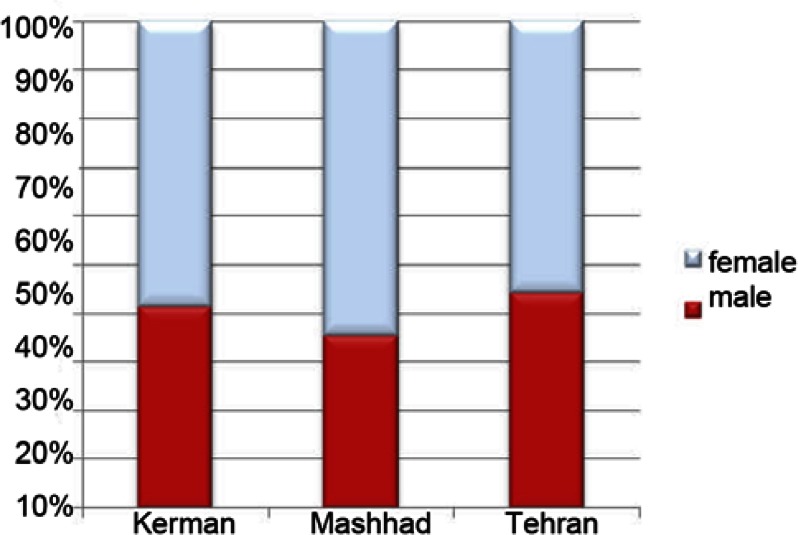
Percentage of genders among patients referred to endodontic offices in three different cities of Kerman, Tehran, and Mashhad

Mean (standard deviation) of age in Kerman patients were 33.80 (11.8) years, in Mashhad patients were 35.22 (14.4) years, in Tehran patients were 36.8 (14.2) years. In all three cities most patients were females (Kerman: 58.6%, Mashhad: 64.4%, Tehran: 55.8%, (Figure 1).

**Figure 2.**
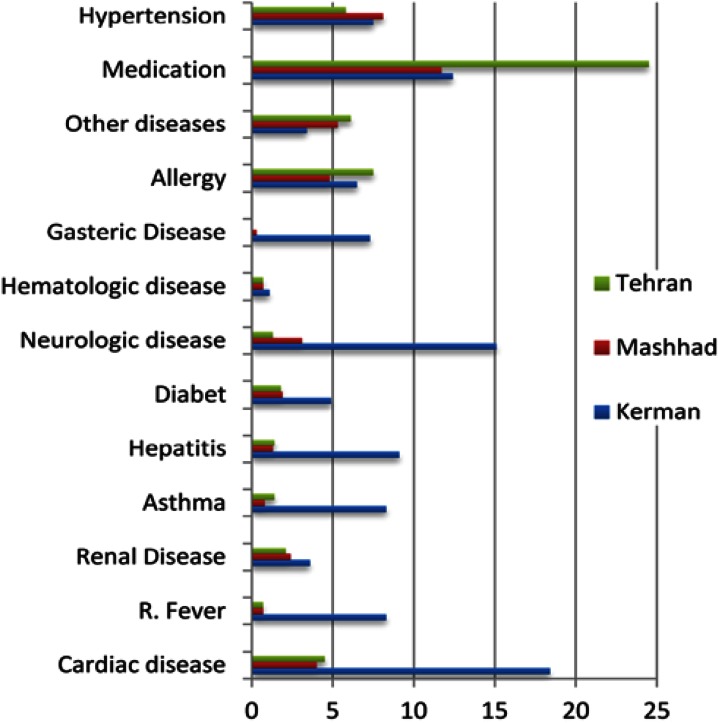
Percentage of various diseases in three cities of Kerman, Tehran, and Mashhad

The most common systemic diseases in Kerman were cardiac disease (18.4%), neurologic disorders (15.1%), and hepatitis (9.1%); while in Mashhad were hypertension (8.1%), allergy (4.77%), and cardiac diseases (3.97%). The most common diseases in Tehran were allergy (7.1%), hypertension (5.84%), and cardiac diseases (4.48%) (Figure 2). Overall, there was greater frequency of patients with systemic diseases referred to the Kerman endodontic office (55.03%) compared to Mashhad (24.32%) and Tehran (22.16%) endodontic offices (P<0.001); however, there was no significant differences in the frequency of patients with systemic diseases referred to the Tehran and the Mashhad endodontic offices (*P*>0.05).

The frequency of systematic diseases had positive association with age in all cities (*P*<0.05).

## 4. Discussion

The results of the present study show that the frequency of systemic diseases among patients referred to endodontic offices in these three cities are significantly different. Previous investigations mostly focused on the frequency of patients seeking dental treatments in dental schools during a short period of 6 months to 3 years (5, 7-13), whereas the present study has evaluated the frequency of patients referred to the offices limited to endodontic practice during a period of more than 10 years. Sample sizes of the previous investigations were from 571 up to 58317 (5-13). In the present study, documents of 15413 patients have been evaluated.

In the present study self-completed questionnaires which were filled for each patient at the beginning of treatment was evaluated. Previous investigations were based on either self-completed questionnaire or interview with patients (5-13). As a retrospective study, a self-completed questionnaire was used in the present investigation; however it has been shown that these questionnaires underestimate the frequency of systemic diseases among referring patients (14).

Previous investigations on the prevalence of systemic diseases among patients seeking dental and periodontal treatment have shown that with age the prevalence of medical disorders significantly increases (5, 10). The results of the present study have been in accordance with previous investigations on endodontic patients (5, 10, 13). Most of the subjects in this study (patients seeking endodontic care) were female as with previous investigations (8, 10-13).

Results of the present study have shown that while there was no significant differences in the percentage of referred patients with systemic diseases between Tehran and Mashhad, there was a significant difference of the patients with systemic diseases in Kerman endodontic office compared to two other cities (*P*<0.05). It should be considered that dentists in smaller cities might have a tendency to refer patients with systemic diseases to the endodontists. Another reason might be due to the n umber of endodontist in relation to total population of these cities. Kerman has the lower population compared to Tehran and Mashhad and their dentists would rather refer their patients with systemic diseases to the endodontists instead of treating them by themselves.

In Iran most of the postgraduate students have to start their practice following graduation in small cities. The results of the present study have shown that preparing them for encountering high frequency of patients with systemic diseases should be emphasized during postgraduate programs. Moreover, in continuing education programs management of patients with systemic diseases should be reflected to respond growing trend of patients with systemic disorders seeking dental treatment.

The results of the present study regarding significantly higher frequency of patients with systemic diseases referring to the endodontic offices in small cities have shown that not only postgraduate curriculum of endodontics should be changed to prepare the postgraduate students for future patients demand with systemic diseases but also other related postgraduate programs should be reevaluated.

Previous investigations in various geographic places have reported different frequency of systemic diseases among patients seeking dental treatments (5-13). The results of the present study have shown that even in one country the frequency of systemic diseases among patients referring to the offices limited to endodontic practices were different.

The results of the present study have shown that two cities with higher population and similar economic standing (Mashhad and Tehran) show similar frequency of certain systemic diseases (allergy, hypertension, and cardiac diseases) among three more common systemic diseases while patient in Kerman reported more neurologic disorders, and hepatitis (mostly hepatitis A). Based on latest available data cardiovascular problem is one the most common reason of death among Iranian population and therefore, the results of the present study show that more focus should be made on postgraduate programs to prepare postgraduate students for managing patient with cardiac diseases.

## 5. Conclusion

Based on our results the frequency of medically compromised patients and the type of systemic diseases refer to an endodontic office might be different in each city and postgraduate students should be prepared for managing patients with various medically compromised conditions. Also, other postgraduate programs such as periodontics, prosthodontics, and restorative dentistry curriculum should be reevaluated as these patients will be referred to them following completion of their endodontic treatment.
